# Digital Normativity: A Challenge for Human Subjectivation

**DOI:** 10.3389/frai.2020.00027

**Published:** 2020-04-28

**Authors:** Eric Fourneret, Blaise Yvert

**Affiliations:** Inserm and Univ Grenoble Alpes, BrainTech Lab U1205, Gières, France

**Keywords:** artificial intelligence, machine learning, free will (freedom), agency, ethics, education, normativity, governance

## Introduction

Recent advances in artificial intelligence (AI) have opened unprecedented opportunities to humans to think and operate the world and its increasing complexity with digital technologies. Striking examples are deep neural networks (DNNs) (Lecun et al., 2015; Mnih et al., 2015), which can be trained quickly on large datasets either self-generated or already available from human experience. In particular, algorithms can become more efficient than humans on specific tasks after relatively short training periods compared to the time that humans need to learn (few hours or days, as compared to years). Their technical efficiency has for instance been demonstrated for optimizing financial transactions, speech or text recognition (Hinton et al., 2012), language translation (Hassan et al., 2018), real-time image content analysis, autonomous driving (Chen et al., 2015), or playing chess or go (Silver et al., 2017). They also start to see use in medicine to reach diagnoses (Lehman et al., 2019; Ye et al., 2019) and improve neuroprosthetics (Bocquelet et al., 2016; Schwemmer et al., 2018; Anumanchipalli et al., 2019). This multiplicity of technical demonstrations is thus progressively bringing AI central and ubiquitous in human life. Yet, the effectiveness of algorithms in bringing more and more relevant recommendations to humans may start to compete with human-alone decisions based on values other than pure efficacy. Here, we examine this tension in light of the emergence of several forms of digital normativity, and analyze how this normative role of AI may influence the ability of humans to remain subject of their life.

## The Advent of Digital Normativity

The increasing role of AI is engendering the emergence of several forms of digital normativity, the ability of algorithms to establish standards that humans incorporate as what should be considered as normal in their lives and guide their actions. First, algorithms tend to reproduce the trends that are most present in the data on which they have been trained. This creates a normalized view of the problem they are intended to solve. The level of details that algorithms might be able to discriminate can be high, as for instance, in automated image pattern recognition or autonomous driving (Kaur and Rampersad, 2018). This first form of digital normativity may thus often be satisfying enough for humans to rely on algorithmic recommendations. However, the automatic and thus objective processing of large datasets restitutes general trends present in these datasets, whether ethically good or bad (Hardt et al., 2016).

Another form of digital normativity arises from the use of predictive algorithms trained on objective observational data without accounting for the course through which this data has been generated. For instance, algorithms that provide a customer with purchasing suggestions only rely on previous purchases made by the same and other customers, without access to the personal reasons underlying these purchases. This form of automatic data processing thus eliminates the inherent subjectivity of the customer: The individual is objectivized (normalized) by the algorithm (Ayres, 2007). This second form of digital normativity is actually a recursive and dynamic process: Algorithmic recommendations emanating from previous human actions in turn influence their next actions (Rouvroy and Berns, 2013; Thomassey and Zeng, 2018).

The normative role of algorithms takes a third form when their efficiency outperforms that of humans. If, for a given application, an algorithm has a higher predictive power than any human expert, it may indeed become reasonable to rely solely on this algorithm to make decisions. The algorithm then creates the norm by imposing its efficacy. The efficiency becoming the norm, the question becomes whether the role of humans in determining for themselves the finality of this efficiency could be challenged.

## Digital Normativity and Subjectivation

Subjectivation (Wieviorka, 2012) is a construction process leading someone to become and be aware of being a subject, i.e., being free and responsible for one's actions and at the foundation of one's representations and judgments. This capacity is progressively acquired throughout life experience, including education, professional life, and more. Given that AI now constitutes an important part of human environment, could this technology weaken, or on the contrary, help to boost the capacity of human individuals to become subjects of their individual and collective lives?

Such question could be considered as irrelevant since it is humans who develop AI algorithms. This role ensures that the human action remains required, and if algorithms help to make decisions, their recommendations still result from a set of rules established by humans. However, AI algorithms may still influence the process of subjectivation. For instance, a search engine giving access to a huge amount of available knowledge in just a few clicks offers unique opportunities to any individual to build his or her critical judgment, and thus to become a human subject. The same engine may also bias subjectivation when results put at the top of the list are based on a statistical inference that does not account for the user as a subject.

Once subjectivation has been acquired, AI may further influence how it is exerted. Humans may indeed no longer desire to make decisions by themselves whenever algorithms may efficiently handle for them this task. This could be for the sake of either physical comfort when an action is physically demanding (e.g., driving long distances), or psychological comfort when a decision engages a moral responsibility difficult to endorse. As such, algorithms are used in the justice system in Belgium to evaluate the risk of recidivism and help determine whether an imprisoned individual should benefit from anticipatory freedom. In this scenario, the judges' responsibility may be increased and more difficult to stand if they decide against the recommendation of an algorithm. If their decision is indeed found later to be inappropriate, they could be opposed to have acted against an algorithmic decision considered more objective than a human decision (Rouvroy and Berns, 2013). Although it remains theoretically possible to resist such normativity, the associated amplification of human responsibility could become so much of a deterrent that disobedience would become difficult or even no longer possible in practice. An increasing number of opportunities may therefore be offered to humans to progressively disengage from their role of subjects of their lives (Erel et al., 2019), leading to the emergence of certain forms of governance without subject.

## The Risk of a Silent Human Desubjectivation

Despite their importance in the organization of human societies, algorithms do not decide alone and a cooperative relationship between humans and AI exists: on the one hand a form of expertise (the algorithm) and on the other hand the power to decide (humans). Each needs the other but both do not merge as one. Indeed, a competence to make decisions differs from a competence of expertise: A power to decide can be exerted in absence of expertise, and conversely, an expert is not necessarily competent to decide (Green, 2012; Heitz, 2013). Deciding is acting with doubts, thus accepting the risk of making errors. If humans were to refuse this risk and transfer their power of decision to more efficient algorithms, they would jeopardize an essential part of their humanity: their ability to learn from errors and thus their power of perfectibility (Rousseau, 1754).

Current generations remain vigilant regarding this risk but what about future generations born after the emergence of digital normativity, and thus well-habituated with its ubiquity? When introducing the notion of voluntary servitude, La Boétie already seized this question to understand the foundations of despotic political powers. He pointed out that in a process of oppression, people are at first aware of losing their freedom but that the next generations make this situation of oppression the rule and become unaware of their servitude or accustomed to it: “(…) Those who come after serve without regret, and willingly do what their predecessors had done by constraint” (La Boétie, 1576). Importantly, the advent of AI governmentality would not impose itself by any violent physical or moral means, but by meddling into human life through progressive changes of practice. This is where a risk of silent human desubjectivation could take root.

This risk is further strengthened by the challenge of explicability of AI algorithms. Although the methods used to train algorithms are well-understood (e.g., backpropagation), the resulting set of optimal parameters does not generally represent any intuitive or ecological meaning for a human being. Then the question is: Can we ethically follow a recommendation deduced through a reasoning surpassing human expertise but no longer accessible? The risk would be to make decisions blindly without critical evaluation, thus silencing the capability of the human subject to distinguish between the fair and the unfair.

## Conclusion: The Necessity of an Ethics by Design

AI has clearly become a unique opportunity for accompanying the evolution of human well-being but engenders a new major ethical challenge for humans: to preserve our capability to remain subjects and not only agents. Far from either completely embracing or completely rejecting AI technologies, it has become essential that an ethical reflection accompany the current developments of intelligent algorithms beyond the sole question of their social acceptability. Such thoughtful reflection cannot be conducted independently from the scientific actors of AI technology, but needs to accompany them in defining the values and aims of their research. The Ethics-by-design methodology introduced by Verbeek (2011) can be used for such purpose. When designing a new technology, this methodology consists first in identifying the system of values of the technology (e.g., the power of objective prediction of AI and its efficiency in extracting relevant features of massive amount of data), and then in thinking the principles of protection of the subjectivation process from the beginning of the conception of the technology (e.g., how a speech neural prosthesis can be conceived in such a way that the externalization of the user's inner speech remains under his full control, Rainey et al., 2018). In practice, ethics by design can be implemented by anchoring philosophers and ethicists in scientific groups developing the technologies. Moreover, educational programs toward the next generations of scientists born with AI and dedicated to the ethical implications of AI would also be key elements to ensure the perenity of such ethical reflection. This double scientific and societal anchoring of a pragmatic ethics is mandatory to preserve human subjectivation, free will, and freedom in the long term: “Techniques always bring with them the world in which they will make sense” (Guchet, 2014). AI should not be developed to invent the future for us, but to help us invent our future.

## Author Contributions

EF and BY conducted this reflection and wrote the manuscript.

## Conflict of Interest

The authors declare that the research was conducted in the absence of any commercial or financial relationships that could be construed as a potential conflict of interest.

## References

[B1] Anumanchipalli G. K. Chartier J. Chang E. F. (2019). Intelligible speech synthesis from neural decoding of spoken sentences. Nature 568, 493–498. 10.1038/s41586-019-1119-1 31019317 PMC9714519

[B2] Ayres I. (2007). Super Crunchers. Why Thinking-by-Numbers is the New Way to be Smart. Bantam; Reprint Edition.

[B3] Bocquelet F. Hueber T. Girin L. Savariaux C. Yvert B. (2016). Real-time control of an articulatory-based speech synthesizer for brain computer interfaces. PLOS Comput. Biol. 12:e1005119. 10.1371/journal.pcbi.1005119 27880768 PMC5120792

[B4] Chen C. Seff A. Kornhauser A. Xiao J. (2015). “DeepDriving: learning affordance for direct perception in autonomous driving,” in Proceeding IEEE ICCV, 2722–2730. 10.1109/ICCV.2015.312

[B5] Erel I. Stern L. H. Tan C. Weisbach M. S. Selecting Directors Using Machine Learning (2019). Fisher College of Business Working Paper No. 2018-03-005, Finance Working Paper No. 605/2019. European Corporate Governance Institute (ECGI). Available online at: https://ssrn.com/abstract=3144080

[B6] Green C. (2012). Nursing intuition: a valid form of knowledge. Nurs. Philos. 13, 98–111. 10.1111/j.1466-769X.2011.00507.x 22405017

[B7] Guchet X. (2014). Philosophie des Nanotechnologies. HR.EVOL.MA., ed. Paris:Hermann Paris.

[B8] Hardt M. Price E. Srebro N. (2016). “Equality of opportunity in supervised learning,” in Proceeding NIPS, 3323–3331.

[B9] Hassan H. Aue A. Chen C. Chowdhary V. Clark J. Federmann C. (2018). Achieving human parity on automatic chinese to english news translation. arXiv[Preprint].arXiv:1803.05567.

[B10] Heitz J. (2013). La décision : ses fondements et ses manifestations. RIMHE 1, 106–117. 10.3917/rimhe.005.0106

[B11] Hinton G. Deng L. Yu D. Dahl G. Mohamed A. R. Jaitly N. (2012). Deep neural networks for acoustic modeling in speech recognition: The shared views of four research groups. IEEE Signal Process. Mag. 29, 82–97. 10.1109/MSP.2012.2205597

[B12] Kaur K. Rampersad G. (2018). Trust in driverless cars: investigating key factors influencing the adoption of driverless cars. J. Eng. Tech. Manag. 48, 87–96. 10.1016/j.jengtecman.2018.04.006

[B13] La Boétie E. (1576). Discours de la servitude volontaire ou le Contr'un.

[B14] Lecun Y. Bengio Y. Hinton G. (2015). Deep learning. Nature 521, 436–444. 10.1038/nature14539 26017442

[B15] Lehman C. D Yala A. Schuster T. Dontchos B Bahl M. Swanson K.. (2019). Mammographic breast density assessment using deep learning: Clinical implementation. Radiology 290, 52–58. 10.1148/radiol.2018180694 30325282

[B16] Mnih V. Kavukcuoglu K. Silver D. Rusu A. A. Veness J. Bellemare M. G.. (2015). Human-level control through deep reinforcement learning. Nature 518, 529–533. 10.1038/nature14236 25719670

[B17] Rainey S. Maslen H. Megevand P. Arnal L. Fourneret E. Yvert B. (2018). Neuroprosthetic speech: the ethical significance of accuracy, control and pragmatics. Cambridge Q. Healthc. Ethics. 28, 657–670. 10.1017/S0963180119000604 31475659

[B18] Rousseau J. (1754). Discours sur l'origine et les fondements de l'inégalité parmi les hommes. Œuvres Complètes.

[B19] Rouvroy A. Berns T. (2013). Gouvernementalité algorithmique et perspectives d'émancipation. Réseaux 177, 163–196. 10.3917/res.177.0163

[B20] Schwemmer M. A. Skomrock N. D. Sederberg P. B. Ting J. E. Sharma G. Bockbrader M. A.. (2018). Meeting brain–computer interface user performance expectations using a deep neural network decoding framework. Nat. Med. 24, 1669–1676. 10.1038/s41591-018-0171-y 30250141

[B21] Silver D. Schrittwieser J. Simonyan K. Antonoglou I. Huang A. Guez A.. (2017). Mastering the game of Go without human knowledge. Nature 550, 354–359. 10.1038/nature24270 29052630

[B22] Thomassey S. Zeng X. (eds.). (2018). “Artificial intelligence for fashion industry,” in The Big Data Era, Thomassey (Singapore: Springer) 10.1007/978-981-13-0080-6

[B23] Verbeek P.-P. (2011). Moralizing Technology. Chicago: The University Chicago Press. 10.7208/chicago/9780226852904.001.0001

[B24] Wieviorka M. (2012). Du concept de sujet à celui de subjectivation / dé-subjectivation. FMSH-WP-2012–P-2016.

[B25] Ye W. Gu W. Guo X. Yi P. Meng Y. Han F. (2019). Detection of pulmonary ground-glass opacity based on deep learning computer arti fi cial intelligence. Biomed. Eng. Online 18, 1–12. 10.1186/s12938-019-0627-4 30670024 PMC6343356

